# High prevalence of nonalcoholic steatohepatitis and abnormal liver stiffness in a young and obese Mexican population

**DOI:** 10.1371/journal.pone.0208926

**Published:** 2019-01-04

**Authors:** Maricruz Sepulveda-Villegas, Sonia Roman, Ingrid Rivera-Iñiguez, Claudia Ojeda-Granados, Karina Gonzalez-Aldaco, Luis Alberto Torres-Reyes, Alexis Jose-Abrego, Arturo Panduro

**Affiliations:** Department of Molecular Biology in Medicine, Civil Hospital of Guadalajara, “Fray Antonio Alcalde,” Guadalajara, Jalisco, Mexico and Health Sciences Center, University of Guadalajara, Guadalajara, Jalisco, Mexico; Medizinische Fakultat der RWTH Aachen, GERMANY

## Abstract

**Objective:**

To identify nonalcoholic steatohepatitis (NASH) and liver stiffness in Mexican subjects with different body mass index (BMI).

**Methods:**

A cross-sectional study was conducted in 505 adults. Risk for NASH was defined as the presence of one or more of the following biochemical and metabolic parameters (BMPs): fasting glucose ≥100 mg/dl, triglycerides (TG) ≥150 mg/dl, homeostatic model assessment of insulin resistance (HOMA-IR) ≥2.5, aspartate aminotransferase (AST) >54 IU/L and alanine aminotransferase (ALT) >42 IU/L. Body mass index measurement and nutritional assessment were performed by standard procedures. Liver fibrosis stage was determined by liver stiffness measurement using transitional elastography (TE) or by liver biopsy (LB).

**Results:**

Risk for NASH was 57% (290/505). Most BMPs values incremented by BMI category. Among 171 at-risk patients, 106 subjects were evaluated by TE and 65 subjects by LB. Abnormal liver stiffness (≥6.0 kPa) was prevalent in 54% (57/106) of the cases, whereas by LB, 91% (59/65) of patients with obesity had NASH and liver fibrosis. Furthermore, liver fibrosis was prevalent in 46% (6/13) in normal weight individuals, whereas 4.6% (3/65) of patients with a BMI ≥ 35 kg/m^2^ showed no histopathological abnormalities. Overall, 67.8% (116/171) of the patients had abnormal liver stiffness or NASH. The normal weight patients with liver damage consumed relatively a higher fat-rich diet compared to the other groups whereas the remaining subgroups shared a similar dietary pattern.

**Conclusion:**

Young patients with overweight and obesity showed a high prevalence of altered BMPs related to abnormal liver stiffness assessed by TE and NASH by LB. Early diagnostic strategies are required to detect the risk for NASH and avoid further liver damage in populations with a rising prevalence of obesity by defining the risk factors involved in the onset and progression of NASH.

## Introduction

The main etiologies of chronic liver disease worldwide are chronic alcohol abuse, viral hepatitis B and C followed by nonalcoholic fatty liver disease (NAFLD) [1]. Regional variances in the epidemiology of these etiologies have been documented [2]. For example, in high-resource countries, alcoholic liver disease has decreased in the last decade [3], whereas the new era of direct-acting antivirals promises near-future eradication of hepatitis C virus [4,5]. However, these tendencies may not occur in low-resource countries [6,7].

In contrast, NAFLD including simple steatosis and non-alcoholic steatohepatitis (NASH) has become a global trend in parallel to the uprising rate of obesity in populations that have acquired a Westernized lifestyle [8]. Overall, it has been estimated that NAFLD affects 25.24% of the world population with significant differences between Africa and the Western World (13–30%) [8]. Nonetheless, genetic susceptibility can importantly modify the incidence and progression of NAFLD/NASH within populations [8–10].

NASH is a pathophysiological stage activated by the continuous deposition of excess liver triglycerides (steatosis) due to increased dietary fat intake or by *de novo* lipogenesis. It is also characterized by insulin resistance (IR), inflammation and oxidative stress that eventually leads to fibrosis, cirrhosis, and in some cases, liver cancer [11]. Conventionally, NASH diagnostics relies on a liver biopsy (LB) when all other causes of liver damage have been discarded in which hepatocyte ballooning, inflammation, and fibrosis are demonstrated. Alternatively, non-invasive strategies that include liver stiffness measurement (LSM) using transient elastography (TE) [12–14] and other surrogate scores such as ELF, FIB-4, and NFS are used to spare the patient of histology examination [15]. However, LB or some non-invasive diagnostic tools may be unfeasible for screening patients at early stages of disease among the general population who are overweight and obesity, which may also hinder the study of the natural history of NASH.

Regarding this point, 1.9 billion people are overweight, and 650 million people are obese worldwide [16]. The relationship between obesity, hepatic steatosis, and liver inflammation is evident as shown in a recent meta-analytic global assessment study that reported a pooled overall obesity prevalence of 51.34% (95% CI 41.38–61.20) in NAFLD patients and 81.83% (95%CI 55.16–94.28) among NASH patients [17]. However, in the context of obesity, some patients with fatty liver may not develop NASH, while others who are lean could develop fibrosis. These differences may be related to the population´s genetic and lifestyle risk factors that affect the rate at which these morbidities occur. Therefore, the factors associated with the onset and clinical outcome of NASH should be evaluated by population. For example, in Mexico, the Amerindian-European-African admixture of the population and the consumption of an industrialized diet are risk factors involved in the national obesity epidemic [18–20], dyslipidemias and cardiovascular disease [21–24]. Currently, 72.5% of the Mexican population is overweight or obese, while 2.9% are extremely obese [25]. However, to date, nationwide epidemiological studies regarding the prevalence or the risk for NASH in the Mexican population are lacking [26,27].

NAFLD, NASH and type 2 diabetes are diseases linked to the pathogenic context of obesity. Therefore, all these pathophysiological conditions share several altered metabolic and biochemical parameters (BMPs) such as hyperglycemia, dyslipidemia, mainly hypertriglyceridemia (HTG), and IR [28–30]. Also, elevation of liver aspartate aminotransferase (AST) and alanine aminotransferase (ALT) is associated with liver inflammation or hepatocellular injury [31]. Thereby, this study aimed to detect patients with risk of NASH by using these BMPs and assess liver stiffness in subjects with different BMI.

## Material and methods

### Study protocol

A cross-sectional study was carried out from August 2011 to September 2015 at the Nutrigenetic Clinic of the Department of Molecular Biology in Medicine, Civil Hospital of Guadalajara, “Fray Antonio Alcalde” which is open to the general population for nutritional consultation. This hospital facility attends the most socially and economically vulnerable people who have a low income, a situation that is common in at least 70% of the Mexican population [25].

The study group included 505 unrelated, apparently healthy adults from the general population, male and female ≥18 years with a BMI ≥18.5 kg/m^2^. Patients were without a previous diagnosis of NAFLD/NASH or any other liver pathology. Exclusion criteria were subjects with a history of significant alcoholic consumption (>20 g/day and >40g/day for females and males, respectively) and acute or chronic viral hepatitis B or C assessed by serological, molecular and clinical evaluation. Other causes of liver disease such as hemochromatosis, α-1 antitrypsin deficiency, Wilson’s disease, primary sclerosing cholangitis, primary biliary cholestasis, drug-induced hepatotoxicity or autoimmune liver disease were also discarded using the standard criteria [32]. Patients with hypertension (≥130 mm Hg) or history of stroke were not included to reduce the possibility of comorbidity with cardiovascular disease.

### Body mass index measurement

Height was measured by a stadiometer (Rochester Clinical Research, New York, NY, USA). Body composition was assessed by electrical bio-impedance using an InBody 3.0 instrument (Analyzer Body Composition, Biospace, Seoul, Korea) which reflected total weight, body fat, and BMI. Subjects were classified according to BMI (kg/m^2^) based on the criteria of the World Health Organization: Normal weight (NW) (18.5–24.9 kg/m^2^), Overweight (OW) (25–29.9 kg/m^2^), Obesity I (OB I) (30–34.9 kg/m^2^), Obesity II (OB II) (35–39.9 kg/m^2^) and Obesity III (OB III) (≥40 kg/m^2^) [33].

### Dietary assessment

Dietary intake was measured by a three-day food record (two weekdays and one weekend day) which has been previously used for our population [20]. The food records were coded using a specialized software (Nutrikcal VO, Mexico) containing a Mexican food database. The total amount of calories, fat, carbohydrates, proteins as well as fiber and cholesterol were calculated.

### Laboratory tests

Blood samples (10 mL) were drawn by venipuncture after a 12-hour overnight fast from the 505 subjects. Biochemical tests included glucose, insulin, triglycerides (TG), total cholesterol (TC), high-density lipoprotein cholesterol (HDL-c), AST, ALT, and gamma-glutamyl transferase (GGT). All biochemical tests were determined with the AU5800 Clinical Chemistry System (Beckman Coulter´s Inc. CA, USA). Low-density lipoprotein cholesterol (LDL-c) was calculated by the Friedewald formula [34]. The very low-density lipoprotein cholesterol (VLDL-c) concentration was calculated as Total Cholesterol—(LDL-c + HDL-c). Prediabetes was diagnosed if fasting glucose levels were 100–125 mg/dL and diabetes if fasting glucose levels were ≥126 mg/dL [35]. The homeostatic model assessment of insulin resistance (HOMA-IR) was calculated with the equation: IR = fasting plasma glucose (mg/dL) x fasting serum insulin (μU/mL)/405 [36].

### Definition of risk for NASH

The study population was stratified into two groups: non-risk and at-risk for NASH. Subjects at risk for NASH were defined as those who presented at least one of the following biochemical and metabolic parameters (BMPs): fasting glucose ≥100 mg/dL, TG ≥150 mg/dL, HOMA-IR ≥2.5, AST >54 IU/L and ALT >42 IU/L [29, 30, 37–40].

### Criteria for liver damage assessment

**Fig 1:**
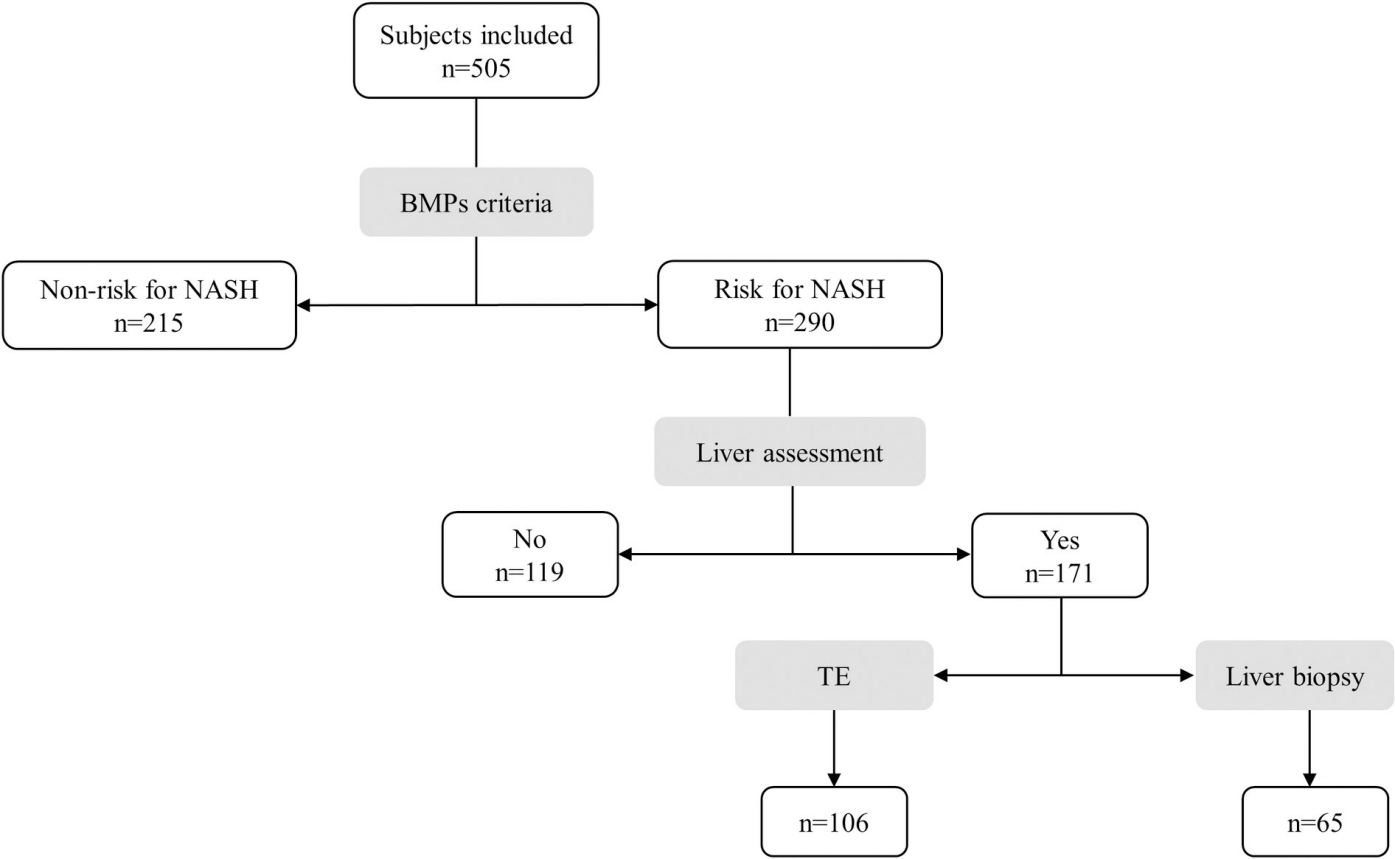
Flow diagram of the study. BMP: biochemical and metabolic parameters; NASH: nonalcoholic steatohepatitis; TE: transient elastography.

As shown in the workflow chart (Fig 1), patients allocated in the at-risk group were evaluated for liver damage either by LSM using TE or LB.

### Liver stiffness measurement and fibrosis staging

LSM was performed using a TE instrument (FibroScan Echosens, Paris, France) with the XL probe in all analyzed patients to stage liver fibrosis. The average values of ten successful readings were used as an indicator of liver stiffness expressed in kilopascals (kPa). Liver fibrosis was staged as follows: F0: <6 kPa (no fibrosis), F1: 6.1–7.0 kPa (initial fibrosis), F2: 7.1–8.8 kPa (intermediate fibrosis), F3: 8.9–11.8 kPa (advanced fibrosis) and F4: ≥11.9 kPa (liver cirrhosis) [12].

### Liver biopsy and histological examination

Liver biopsy was performed in the patients with a BMI ≥ 35 kg/m^2^ who underwent bariatric surgery at the Surgery of Obesity and Metabolic Diseases Clinic. Liver biopsies were obtained as a wedge biopsy by the surgeon medic. All liver specimens were fixed in a 4% formalin solution, embedded in paraffin and stained with Periodic Acid-Schiff (PAS), Hematoxylin-Eosin (H&E) and Masson´s trichrome. The biopsy samples were analyzed independently by two experienced pathologists blinded to the clinical data. All samples were not less than 15 mm long and showed not less than ten portal tracts.

Histopathological diagnosis of NASH was achieved according to the criteria of Brunt *et al*. [40] Liver steatosis was defined according to the percentage of cells with fatty droplets, graded as G0 (<5%); G1 (5–33%); G2 (>33–66%) and G4 (>66%). The degree of necroinflammation was evaluated by the histological features of NASH (steatosis, ballooning, and intra-acinar and portal inflammation), and divided into three categories. Grade 1 (mild) is a NASH minimum criteria diagnosis, involving macrovascular steatosis, occasional ballooned zone 3 hepatocytes, and lobular mixed inflammation (acinar and portal); Grade 2 (moderate) is any degree of steatosis, the visible presence of hepatocytic ballooning and disarray predominantly in zone 3 associated with pericellular fibrosis, and mild to moderate portal inflammation; Grade 3 (severe) is persistent ballooning and disarray of mainly zone 3, increased lobular and portal inflammation compared with grade 2.

The pattern of fibrosis reflects the rate of progression, deposition of connective tissue and architectural remodeling, which was rated on a 0–4 scale: F0, absence of fibrosis; F1, perisinusoidal/pericellular fibrosis in zone 3; F2, zone 3 perisinusoidal/pericellular and periportal fibrosis; F3, focal or extensive bridging in zone 3 perisinusoidal and portal fibrosis; and F4, cirrhosis [41].

### Statistical analyses

The Kolmogorov-Smirnov test was used to determine if the quantitative variables had a normal distribution. Continuous variables were reported as the mean ± standard deviation (SD), and categorical variables were shown as a percentage (%). Comparative analysis was carried out by Student’s t and chi-square tests, as well as ANOVA to assess differences between groups when appropriate. Statistical analyses were computed using Epi Info 7.1.2.0 (CDC, Atlanta, USA) and IBM SPSS statistics, version 21.0 for Windows (IBM Corp, Inc., Chicago, IL, USA). A *p*-value < 0.05 was considered statistically significant.

### Ethical guidelines

The study complied with the ethical guidelines of the 2013 Declaration of Helsinki. This study was revised and approved by the Ethical Committees of the Civil Hospital of Guadalajara ID#HC141/09. All patients signed an informed consent form before participating in the study.

## Results

### Demographic and clinical characteristics of the study population

As shown in Table 1, the mean age of the study group was 37.1± 13.5 years. Based on the criteria mentioned above, 57% (290/505) of the study population was at risk for NASH. Comparatively, the patients in this group were older than those in the non-risk group (39.5 ± 13 *vs*. 33.9 ± 13.5 years, p = 0.0021) and were more obese (average BMI 32.7 ± 9.3 *vs*. 25.2 ± 6.2 kg/m^2^, p = 0.0012).

**Table 1:** Demographic and clinical characteristics of the study population.

Variable	Total	No risk for NASH	Risk for NASH	*p*-value
Number of subjects (%)	505 (100)	215 (43)	290 (57)	-
Age (years)	37.1±13.5	33.9±13.5	39.5±13	0.0021
Gender F/M (%)	71/29	77/23	68/32	0.2800
BMI (kg/m^2^)	29.5±9	25.2±6.2	32.7±9.3	0.0012
Glucose (mg/dl)	91.9±19.6	84.4±7	97.4±23.7	0.0004
Insulin (μU/ml)	11.7±12.1	6.1±2.7	16.1±14.6	0.0033
HOMA-IR	2.8±3.4	1.3±0.6	4±4.1	0.0051
TC (mg/dl)	186.8±41.4	174.8±32.4	195.7±44.9	0.0001
HDL-c (mg/dl)	45.1±16.8	49.6±19	41.8±14.2	0.0033
LDL-c (mg/dl)	113.3±36.1	108.4±30.6	116.8±39.4	0.0018
VLDL-c (mg/dl)	30.1±26.9	18.7±7.4	38.8±32.5	0.0022
TG (mg/dl)	147.9±129.5	92.1±28.2	189.9±157.2	0.0017
AST (IU/L)	28.7±22.7	22.2±6.4	33.6±28.5	0.0004
ALT (UI/L)	30.4±24.7	21±8.3	37.4±30.1	0.0070
GGT (UI/L)	28.5±21.6	18.1±12.2	36.5±26.8	0.0050

Quantitative values are expressed as mean ± standard deviation unless indicated otherwise. BMI, body mass index; HOMA-IR, homeostasis model assessment of insulin resistance; TG, triglycerides; TC, total cholesterol; HDL-c, high-density lipoprotein cholesterol; LDL-c, low-density lipoprotein cholesterol; VLDL-c, very low-density lipoprotein cholesterol; AST, aspartate aminotransferase; ALT, alanine aminotransferase; GGT, gamma-glutamyl-transferase.

### Biochemical and metabolic parameters of subjects at risk for NASH adjusted by BMI

Among the study group, 290 at-risk subjects for NASH were classified by BMI category as shown in Table 2. Only 22% of the cases were NW whereas the remaining were OW (25%), OB I-II (34%) or OB III (19%). The patients in the OB III group were younger (36.2 ± 11.4 *vs*. 42.2 ± 14.4 years, p = 0.005) than the OW group. No differences were observed in gender. BMPs values incremented as BMI category increased. The patients in the OB III group had the highest levels of glucose (109.6 ± 36.8 mg/dl), insulin (23.9 ±16.8 μU/ml), HOMA-IR (6.9 ± 6.6) and ALT (52.1 ± 31.7 IU/L) compared to NW and OW (*p*<0.05). In contrast, decreased concentrations of lipids were observed in the OB III group with a significant difference in TC (177.3 ± 36.4 mg/dl) and LDL-c (100.3 ± 38.9 mg/dl) levels when compared to OW and OB I-II (*p*<0.05).

**Table 2:** Biochemical and metabolic parameters in subjects with risk for NASH by BMI (*n* = 290).

Variable	NW	OW	OB I-II	OB III	*p*-value
Number of subjects (%)	64 (22)	72 (25)	99 (34)	55 (19)	-
Age (years)	36.4±12.4	42.4±14.4	41.2±12.4	36.2±11.4	0.005^a^
Gender F/M (%)	44/20	46/26	72/27	34/21	0.466
BMI (kg/m^2^)	22.8±1.6	27.6±1.4	34±2.6	48.3±6.9	0.005^b^
Glucose (mg/dl)	86.6±9.7	94.9±13.4	100.5±22.2	109.6±36.8	0.006^c^
Prediabetes (%)	7 (11)	19 (26)	33 (33)	13 (24)	0.007^d^
Type 2 diabetes (%)	0	2 (3)	9 (9)	9 (16)	0.001^c^
Insulin (μU/ml)	11.4±9.4	16.1±17.9	14.6±8	23.9±16.8	0.003^e^
HOMA-IR	2.5±2.2	3.8±3.2	3.8±2.8	6.9±6.6	0.007^e^
TC (mg/dl)	191.7±34.4	201.9±36.7	204±56.5	177.3±36.4	0.002^f^
HDL-c (mg/dl)	45.9±12	41.2±12.2	40.9±17.9	39.3±10.3	0.080
LDL-c (mg/dl)	114.7±28	120.7±37.5	124.8±44.8	100.3±38.9	0.006^f^
VLDL-c (mg/dl)	31.4±15.3	43.2±36.3	42.4±40.9	34.8±21.5	0.087
TG (mg/dl)	157.2±76.2	206±170.4	208.4±200.9	173.2±106.8	0.140
AST (IU/L)	33.8±26	29±21.5	30.4±16.9	45±37.5	0.118
ALT (UI/L)	35.6±24.1	29.2±15.8	36.6±23.1	52.1±31.7	0.001^f^
GGT (UI/L)	32±29.7	25.1±15.5	47.7±33.6	37.4±25.7	0.188

Quantitative values are expressed as mean ± standard deviation unless indicated otherwise. NW, Normal weight; OW, Overweight; OB I, Obesity I; OB II, Obesity II; OB III, Obesity III; BMI, body mass index; HOMA-IR, homeostasis model assessment of insulin resistance; TG, triglycerides; TC, total cholesterol; HDL-c, high density lipoprotein cholesterol; LDL-c, low density lipoprotein cholesterol; VLDL-c, very low density lipoprotein cholesterol; AST, aspartate aminotransferase; ALT, alanine aminotransferase; GGT, gamma-glutamyl-transferase.

^a^OB III *vs*. OW

^b^OB III *vs*. all groups

^c^OB III *vs*. NW and OW

^d^. All groups *vs*. NW

^e^OB III *vs*. NW and OB I-II

^f^OB III *vs*. OW and OB I, II.

### Assessment of liver damage by TE or biopsy

**Fig 2:**
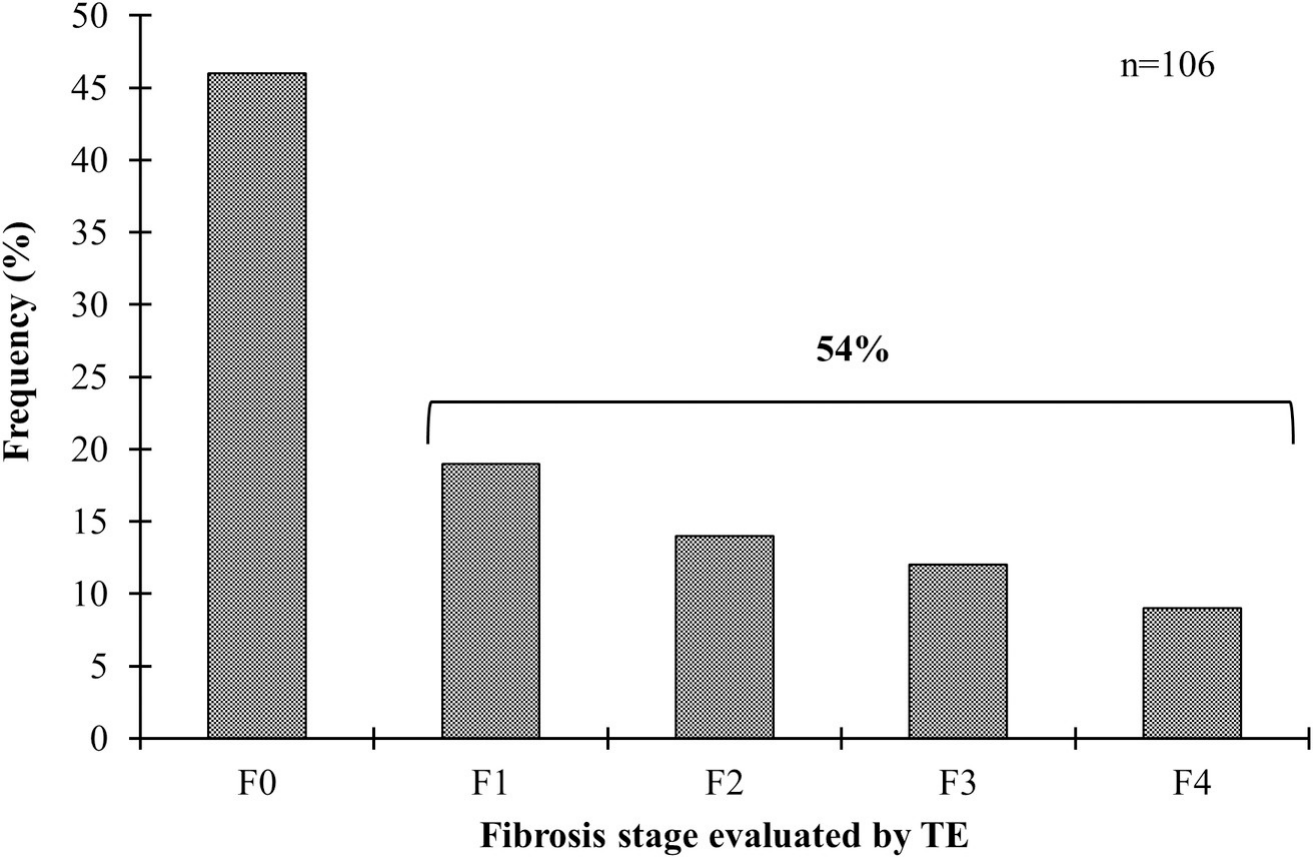
Stage of fibrosis based on LSM by using TE. F1: initial fibrosis; F2: intermediate fibrosis; F3: advanced fibrosis; F4: liver cirrhosis.

TE was performed successfully in 106 subjects. Among these, 46% (49/106) were classified as F0, while abnormal liver stiffness (F1-F4) was found in 54% (57/106) of the cases. The distribution of the stage of fibrosis was F1 35% (20/57); F2, 26% (15/57); F3, 23% (13/57); and F4, 16% (9/57) (Fig 2).

**Fig 3:**
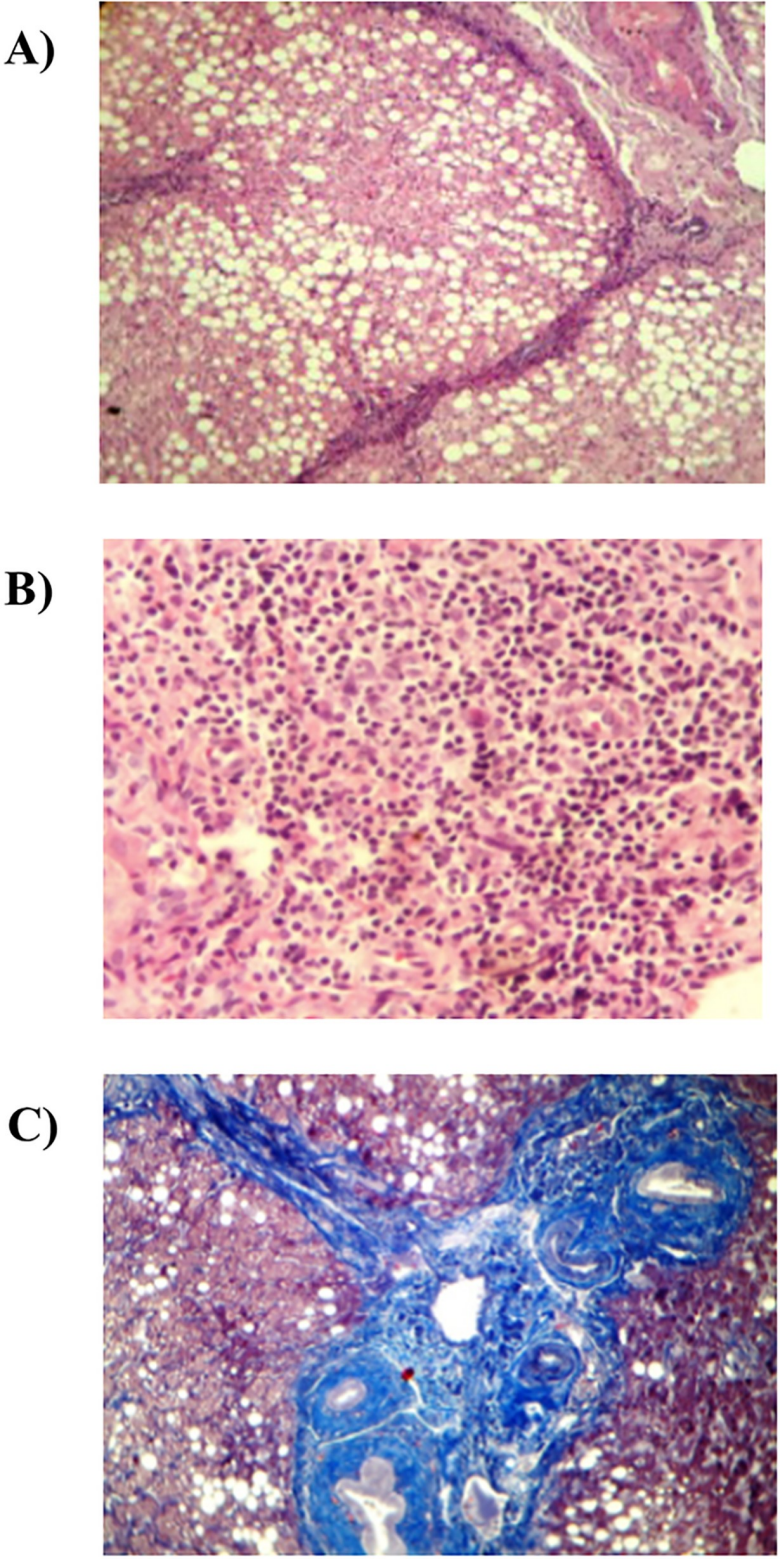
Representative images of histological staining of liver biopsy. A) Steatosis grade was evaluated using Periodic Acid-Schiff (PAS). B) The necroinflammatory grading comprised the presence of steatosis, ballooning, and inflammation evaluated by PAS and H&E. C) Masson´s trichrome stain was used to reveal fibrosis and architectural changes.

**Fig 4:**
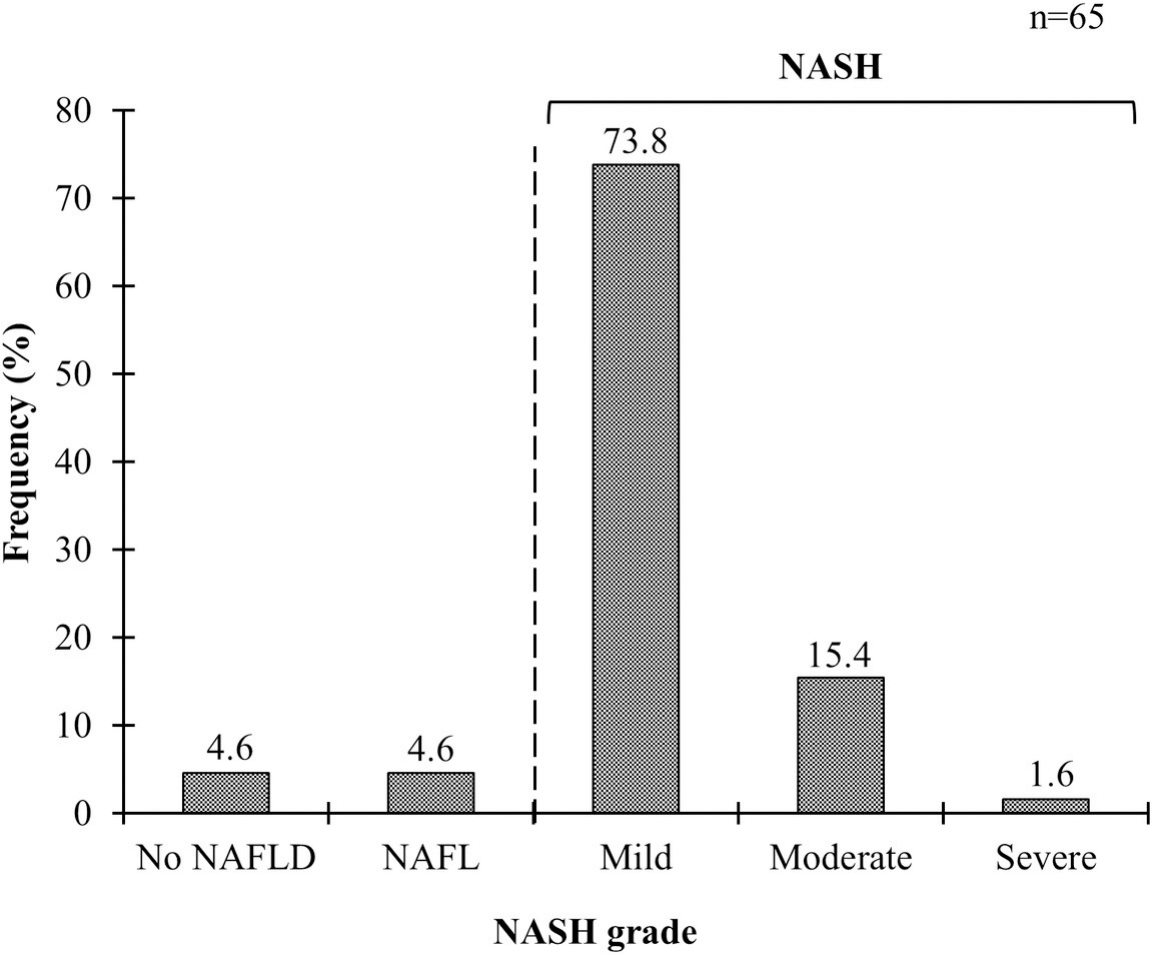
Grading of NASH according to histopathological findings. NAFLD, nonalcoholic fatty liver disease; NAFL: nonalcoholic fatty liver; NASH, nonalcoholic steatohepatitis.

**Fig 5:**
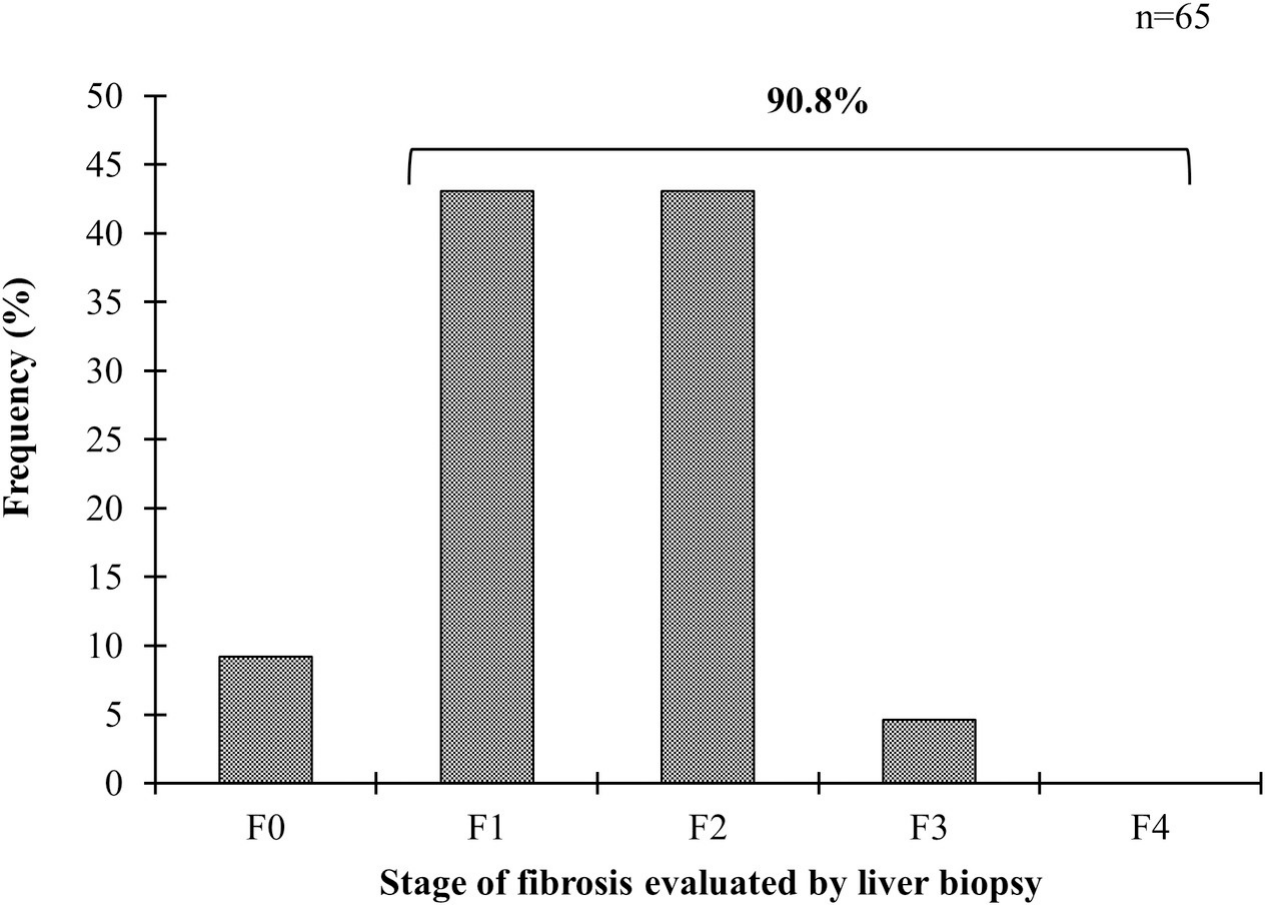
Stage of fibrosis evaluated by liver biopsy. F0: absence of fibrosis; F1: perisinusoidal/pericellular fibrosis in zone 3; F2: zone 3 perisinusoidal/pericellular and periportal fibrosis; F3: focal or extensive bridging in zone 3 perisinusoidal and portal fibrosis; and F4: cirrhosis.

Of the 65 cases evaluated by LB, the histological staining showed steatosis, inflammation, and fibrosis (Fig 3A, 3B and 3C). NASH was prevalent in 90.8% (59/65) of the total cases in which a majority were mild grade (73.8%) whereas NAFLD was absent in 4.6% (3/65) and 4.6% (3/65) had NAFL (Fig 4). Fibrosis staging by LB showed F1 and F2 each in 43.1% (28/65) of the cases, while F3 was found in 4.6% (3/65) (Fig 5).

Table 3 shows the prevalence of liver damage found in the at-risk patients stratified by BMI. Liver damage detected by TE rose from 46% in the NW subjects to 100% in the OB III cases. Additionally, in those evaluated by LB, the presence of NASH with fibrosis was detected in 94% of the OB I-II patients and 90% of the OB III. Overall, the frequency of liver damage assessed either by TE (n = 57) or LB (n = 59) was 67.8% (116/171) among the studied population.

**Table 3:** Prevalence of liver damage detected by TE and LB in patients at risk for NASH stratified by BMI.

	Total 171 (100)	NW 13 (8)	OW 41 (24)	OB I-II 64 (37)	OB III 53 (31)
Subjects evaluated by TE*	106 (62)	13 (12)	41 (39)	48 (45)	4 (4)
Liver stiffness ≥6.0 kPa	57 (54)	6 (46)	18 (44)	29 (60)	4 (100)
Liver stiffness <6.0 kPa	49 (46)	7 (54)	23 (56)	19 (40)	0 (0)
Subjects evaluated by LB*	65 (38)	-	-	16 (25)	49 (75)
NASH	59 (91)	-	-	15 (94)	44 (90)
No-NASH	6 (9)	-	-	1 (6)	5 (10)

* Data is expressed as number (n)/percentage (%).

TE: transient elastography; LB; liver biopsy; BMI: Body mass index (kg/m^2^) by WHO criteria; NW: Normal weight; OW: Overweight; OB I: Obesity I; OB II: Obesity II; OB III: Obesity III; NASH: Nonalcoholic steatohepatitis

In a subset of 21 patients with obesity (BMI >30 kg/m^2^) both TE and LB was performed. Among five cases with an invalid TE result, four had advanced liver fibrosis (>F2) evaluated by LB. In the remaining 16 patients, the stage of liver fibrosis tended to be overestimated by TE in patients who were OB III, whereas those with OB I-II tended to be underestimated in comparison with the LB. (S1 Table).

### Dietary food pattern in patients with liver damage

A nutritional evaluation was conducted in 106 patients within the at-risk group to identify the differences in their dietary food pattern between those who did not have liver damage and those with liver damage. Both groups had an inadequate distribution of macronutrients compared to the reference values. However, patients with liver damage showed significant differences in the consumption of proteins, carbohydrates, and cholesterol (S2 Table). Furthermore, as shown in Table 4, specific differences were found in the distribution of macronutrient consumption in patients with liver damage (n = 64) adjusted by BMI. Patients who were NW consumed a fat-rich diet compared to patients who were either OW or OB (p = 0.009), whereas those who were OB had an excess energy intake related to higher consumption of proteins, total fat, cholesterol, and carbohydrates.

**Table 4:** Comparative dietary food pattern in patients with liver damage based on BMI (n = 64).

Variables	Ref value	NW	OW	OB I-II	OB III	p-value
Subjects, *n* (%)	-	5 (8)	16 (25)	28 (44)	15 (23)	-
Energy, Kcal	-	1884.2**±**585	1965.3±483	2278.9±963	3771.1**±**1954^a^	0.020
Proteins (%)	15%	16.2±2.4	16.8±4.7	17.6±3.5	18.3±4.2	0.659
Total fat (%)	<30%	42±9.7^b^	29.5±9.5	27.8±7.8	31.3±7.8	0.009
SFA (%)	<7%	11.2±4.4	7.4±3.8	7.7±4.1	11.1±4.2	0.053
MUFA (%)	>10%	14.6±9.2	10.4±5.5	8.8±3.3	9.4±4.3	0.545
PUFA (%)	10%	5.8±3.7	4.9±2.1	4.4±1.6	3.9±2.2	0.284
Carbohydrates (%)	55%	45±10	56.4±13.2	56.3±9	51.5±7.5	0.073
Proteins, g	-	77.8±31	81.7±27.2	100.5±48.6	160.1±65.3^c^	0.004
Total fat, g	-	89.7±38.9	62.3±20.3	73.1±43.9	137.8±87.8^d^	0.030
Carbohydrates, g	-	207.2±70.7	281.5±110.5	312.4±116.3	483.1±268.4^e^	0.011
Sugar, g	<50g	43.4±26.9	36.4±27.8	41.6±39.6	43.7±34.8	0.940
Fiber, g	20–30 g	17.7±6.3	25.3±18	21.4±11.3	31.4±21.8	0.185
Cholesterol, mg	<200 mg	440.8±247.4	242.8±200	309.7±204.4	551.2±464.6^d^	0.021

Data are expressed mean ± SD unless indicated. NW, Normal weight; OW, Overweight; OB I-II, Obesity I and obesity II; OB III, Obesity III. Kcal: Kilocalories; SFA: saturated fatty acids; MUFA: monounsaturated fatty acids, PUFA: polyunsaturated fatty acids. Dietary references adapted according to NOM-015-SSA2-2010, NOM-037-SSA2-2012 and ATP III.

^a^OB III *vs*. NW and OW

^b^NW *vs*. OW and OBI I-II

^c^OB III *vs*. NW, OW and OB I-II

^d^OB III *vs*. OW

^e^OB III *vs*. NW.

## Discussion

NAFLD/NASH are currently emerging as primary causes of chronic liver disease, namely cirrhosis, and hepatocellular carcinoma worldwide. While in the era in which effective hepatitis C therapy is a reality and alcohol abuse is being curbed in some populations, the increased prevalence of obesity, type 2 diabetes and NAFLD/NASH affects both the developed and developing countries. The variations in the regional prevalence of these disorders may be related to differences in the underlying genetic and environmental factors that need to be identified appropriately. Therefore, specific population-based diagnostic strategies are required to detect NAFLD/NASH at early stages of progression before advanced liver damage becomes evident.

Thereby, this study was designed to detect patients with risk for NASH in an apparently healthy population based on the fact that hyperglycemia, hypertriglyceridemia, and IR comprise the well-known triad of BMPs altered by obesity and associated with the pathophysiology of NASH [11, 32, 37], whereas elevated values of AST and ALT may reflect liver inflammation and fibrosis [32, 40]. The rationale used in this study allowed us to detect one or more abnormal BMPs in 57% (290/505) of the studied population comprised of relatively young adults (37.1 ±13.5), although at-risk patients were nearly ten years older than those in the non-risk group. Overall, most BMPs increased by BMI category. However, it was notable that 11% of the NW patients had glucose metabolism abnormalities (prediabetes). Insulin and HOMA-IR values progressively increased by BMI category. In contrast, serum TG and cholesterol levels were elevated regardless of BMI category (NW to OB III). On the other hand, liver profile values tended to be lower in patients in the OB III category compared to the other categories except in liver enzymes AST and ALT levels. Overall, these alterations are indicative that liver inflammation and fibrosis processes may have been activated.

In this study, by using both TE and LB, the degree of liver damage at different stages of BMI was detected. Abnormal liver stiffness (≥6.0 kPa) was prevalent in 54% (57/106) of the cases whereas, by LB, 90.8% (59/65) of patients with obesity had NASH and liver fibrosis at stage F1 and F2. In conjunction, liver damage was detected in 67.8% (n = 116/171) of patients with altered BMPs or assuming that those without risk for NASH did not have liver damage in 23% (116/505) of the study population. Interestingly, 46% (6/13) of the NW subjects had liver fibrosis stage F1, and conversely, at the other end of the spectrum, 4.6% (3/65) of the patients with a BMI ≥ 35 kg/m^2^ had a LB without histological evidence of steatosis, inflammation or fibrosis. These findings in young age individuals may be related to genetics, and it has been documented that lean patients can be at risk for NAFLD/NASH [42]. Possible genetic variants may be involved such as *PNPLA3* Ile148 Met [10], and *TM6SF2* Glu167Lys polymorphisms [8,9]. Studies carried out in obese patients from Central Mexico´s population have shown that the *PNPLA3* risk allele (148Met), as well as *LYPLAL*1 and *GCKR* polymorphisms, increased the hepatic triglyceride content in susceptible subjects [27]. As for obesity, it seems that not all patients with increased body weight due to excess fat accumulation will develop liver damage as seen in the OB III group. Further studies are required to elucidate the role of genetics and other factors that may be involved.

It is noteworthy to mention that we cannot discard that TE may have limitations to assess low-grade liver stiffness in lean patients since LSM is known to be more accurate as fibrosis progresses [12, 43]. Nonetheless, TE was useful in most categories of BMI, including those who were OB I-II in which 60% (29/48) had abnormal liver stiffness, whereas LB showed that up to 90% of those who were OB III had NASH. On the other hand, it has been documented that the XL probe is a reliable tool in subjects with significant obesity (≥35 kg/m^2^) [44]. However, we evaluated liver damage with both methods in a subset of 21 obese patients showing differences in the staging of fibrosis. This finding may be related to the fact that excess fat in the thorax and abdominal areas may reduce the accuracy of TE [44], thus favoring the indication of the LB.

The spectrum of metabolic abnormalities and liver damage identified in this study were possibly the result of genetics and unhealthy lifestyles that contribute to obesity-related NAFLD in nations undergoing nutrition transition [45]. In the case of Mexico´s obesity epidemic, in which to date 72.5% are overweight and obese, changes in the dietary pattern have played an important role [25]. In fact, we have defined the regional dietary pattern as a hepatopathogenic diet [19, 46], a feature that predisposes for NAFLD/NASH in susceptible individuals. This type of diet contains more industrialized calorie-dense foods that exceed the recommended amounts of saturated fatty acids, cholesterol, and high-fructose syrups [20, 46] as well as a diminished quantity of polyunsaturated (ω-3/ω-6) fatty acids, fiber, and micronutrients with antioxidant power [47, 48]. In this study, the nutritional assessment showed that NW patients with liver damage consumed relatively a higher fat-rich diet compared to the other groups, and the remaining BMI subgroups shared a similar dietary pattern in which some did not have liver damage as mentioned before. Besides the genes related to fatty liver, these findings may be linked to the ancestral Amerindian-derived risk alleles of the taste receptors TAS1R2, CD36, and TAS2R38 that are known to alter food preferences. These receptors have been associated with dyslipidemia attributed to the consumption of high-fat or high-carbohydrate diets of the Mexican population [18, 49–51]. Therefore, the interactions between dietary profile and genetic susceptibility may mark the difference between those who develop or do not develop liver pathology and risk for NASH, as well as HTG, hypercholesterolemia, and hypoalphalipoproteinemia, the most prevalent dyslipidemias in the Mexican population [21, 32, 46]. Given these underlying conditions, it is important to detect liver damage at early stages and implement nutritional strategies to prevent the rising trend of NAFLD/NASH-related cirrhosis due to excess weight. Healthy diets elaborated with typical Mexican Mesoamerican-derived dishes containing natural food ingredients and traditional cooking procedures provide essential anti-oxidant, anti-inflammatory, and antifibrotic components that may be useful to prevent and manage the progression of NASH [18]. Such foods are not only a healthy alternative; they are also concordant with Mexico´s genetic and food culture background [19].

This study has the following limitations. First, our study was a single-center study prone to selection bias. However, we sought to include patients with typical attributes that are common in more than 70% of the Mexican population. Second, waist circumference, ELF-Score, FIB-4 and gender-specific cut-off values for AST/ALT were not considered. All these auxiliary biomarker/scores for NAFLD/NASH diagnosis have pros and cons, and some do not consider the full spectrum of metabolic risk factors related to NASH. Given the rationale of this work, the five selected BMPs allowed us to detect risk for NASH in all BMI categories among the studied Mexican population who have a specific anthropometric and genetic profile. Nonetheless, we acknowledge that these BMPs may not be extrapolated to other populations. More regional studies are required to determine for every population which biomarkers/scores are best to detect NAFLD/NASH.

In summary, young patients with overweight and obesity showed a high prevalence of altered BMPs related to NASH, abnormal liver stiffness assessed by TE and NASH by LB. Some NW individuals had altered BMPs, prediabetes and liver fibrosis, and several obese subjects had a normal liver. These findings highlight the need for further studies to assess the role of genetics and lifestyle in the onset and progression of NASH. Furthermore, considering the young age of the studied group, periodic monitoring of these patients could aid to improve the quality of life and prevent the appearance of co-morbidities in the next decades of life.

## Supporting information

S1 Table Comparison of liver fibrosis between liver biopsy (LB) and transient elastography (TE) in 21 patients with obesity. (DOCX)

S2 Table Dietary patterns in subjects with risk for NASH (n = 109). (DOCX)
