# Supplementary material for: High prevalence of nonalcoholic steatohepatitis and abnormal liver stiffness in a young and obese Mexican population
Source: PLoS One. 2019 Jan 4;14(1):e0208926. doi: 10.1371/journal.pone.0208926 (PMC6319733; doi:10.1371/journal.pone.0208926)
Supplement: S1 Table — (DOCX) [file pone.0208926.s001.docx]

| **STable 1. Comparison of liver fibrosis between liver biopsy (LB) and transient elastography (TE) in 21 patients with obesity** | | | | | | |
| --- | --- | --- | --- | --- | --- | --- |
| **Staging by LB** | **Staging by TE** | | | | | |
|  | **INVALID** | **F0** | **F1** | **F2** | **F3** | **F4** |
| **F1** | 1 OB III | 4 OB II  1 OB III | - |  | 1 OB III | 3 OB III |
| **F2** | 2 OB III  1 OB II | 2 OB III  1 OB I | - | 1 OB III |  | 3 OB III |
| **F3** | 1 OB III | - | - |  |  |  |
| Obesity I (OB I): IMC 30-34.9 kg/m^2^; Obesity II (OB II): IMC 35-39.9 kg/m^2^; Obesity III (OB III): ≥40 kg/m^2^ | | | | | | |
