# Supplementary material for: High prevalence of nonalcoholic steatohepatitis and abnormal liver stiffness in a young and obese Mexican population
Source: PLoS One. 2019 Jan 4;14(1):e0208926. doi: 10.1371/journal.pone.0208926 (PMC6319733; doi:10.1371/journal.pone.0208926)
Supplement: S2 Table — (DOCX) [file pone.0208926.s002.docx]

**STable 2. Dietary patterns in subjects with risk for NASH (n=109)**

| Variables | **Reference value** | **No liver damage** | **Liver damage** | **P-value** |
| --- | --- | --- | --- | --- |
| Subjects, *n* (%) | - | 45 (41) | 64 (59) | - |
| Energy intake, Kcal | - | 2017.9 ± 625 | 2519.4 ± 1353.7 | 0.011 |
| Proteins (%) | 15% | 16.8 ± 4 | 17.5 ± 3.9 | 0.408 |
| Total fat (%) | <30% | 32.9 ± 9.6 | 30.2 ± 9.0 | 0.130 |
| SFA (%) | <7% | 8.9 ± 3.6 | 8.7 ± 4.3 | 0.852 |
| MUFA (%) | >10% | 11.1 ± 5 | 9.9 ± 4.9 | 0.163 |
| PUFA (%) | 10% | 5.1 ± 2.8 | 4.5 ± 2.1 | 0.485 |
| Carbohydrates (%) | 55% | 52.2 ± 10.8 | 54.3 ± 10.3 | 0.300 |
| Proteins (g) | - | 83.3 ± 30.5 | 108.0 ± 55.8 | 0.004 |
| Total fat (g) | - | 76.6 ± 42.3 | 86.8 ± 59.9 | 0.325 |
| Carbohydrates (g) | - | 256.5 ± 72.7 | 336.5 ± 180.3 | 0.002 |
| Sugar (g) | <50g | 36.8 ± 25.3 | 40.9 ± 34.3 | 0.492 |
| Fiber, g | 20-30 g | 19.0 ± 11.5 | 24.4 ± 16.1 | 0.053 |
| Cholesterol (mg) | <200 mg | 285.6 ± 202.0 | 359.8 ± 305.1 | 0.002 |
| Kcal: Kilocalories; SFA: saturated fatty acids; MUFA: monounsaturated fatty acids, PUFA: polyunsaturated fatty acids. Dietary references adapted according to NOM-015-SSA2-2010, NOM-037-SSA2-2012 and ATP III | | | | |
